# Automated versus conventional perioperative glycemic control in adult diabetic patients undergoing open heart surgery

**DOI:** 10.1186/s12871-022-01721-6

**Published:** 2022-06-16

**Authors:** Roland Kaddoum, Amro Khalili, Fadia M. Shebbo, Nathalie Ghanem, Layal Abou Daher, Arwa Bou Ali, Nour El Hage Chehade, Patrick Maroun, Marie T. Aouad

**Affiliations:** 1 Department of Anesthesiology and Pain Medicine, American University of Beirut Medical Center, Beirut, Lebanon; 2 Faculty of Medicine, American University of Beirut, Beirut, Lebanon

**Keywords:** Glycemic control, Hypoglycemia, Cardiac surgical procedures, Insulins

## Abstract

**Background:**

Intraoperative glycemic variability is associated with increased risks of mortality and morbidity and an increased incidence of hyperglycemia after cardiac surgery. Accordingly, clinicians tend to use a tight glucose control to maintain perioperative blood glucose levels and therefore the need to develop a less laborious automated glucose control system is important especially in diabetic patients at a higher risk of developing complications.

**Methods:**

Patients, aged between 40 and 75 years old, undergoing open heart surgery were randomized to either an automated protocol (experimental) or to the conventional technique at our institution (control).

**Results:**

We showed that the percentage of patients maintained between 7.8–10 mmol.l^−1^ was not statistically different between the two groups, however, through an additional analysis, we showed that the proportion of patients whose glucose levels maintained between a safety level of 6.7–10 mmol.l^−1^ was significantly higher in the experimental group compared to control group, 14 (26.7%) vs 5 (17.2%) *P* = 0.025. In addition, the percentage of patients who had at least one intraoperative hyperglycemic event was significantly higher in the control group compared to the experimental group, 17 (58.6%) vs 5 (16.7%), *P* < 0.001 with no hypoglycemic events in the experimental group compared to two events in the control group. We also showed that longer surgeries can benefit more from using the automated glucose control system, particularly surgeries lasting more than 210 min.

**Conclusion:**

We concluded that the automated glucose control pump in diabetic patients undergoing open heart surgeries maintained most of the patients within a predefined glucose range with a very low incidence of hyperglycemic events and no incidence of hypoglycemic events.

**Trial registration:**

Registered with clinicaltrials.gov (NCT #NCT03314272, Principal investigator Roland Kaddoum, date of registration: 19/10/2017).

## Introduction

Decades ago, glucose control became an important treatment goal in hospitalised patients [1]. The notion of tight glycemic control became more prominent in 2001 when a landmark study by Van Den Berghe demonstrated a significant decrease in mortality when maintaining blood glucose between 4.4 and 5.6 mmol.l^−1^ in intensive care unit patients [1].

It has been found that the incidence of hyperglycemia after cardiac surgery is very high (stress induced hyperglycemia), and it occurs almost universally after cardiac surgeries, regardless of whether a person is diabetic or not [2]. The mechanisms by which hyperglycemia affect outcomes could be related to suppressive effects on immune function and an associated increased risk of infection, endothelial damage, hepatocyte mitochondrial damage, and potentiation of tissue ischemia due to acidosis or inflammation [3–5].

Knowing that hyperglycemia, hypoglycemia, and increased glycemic variability have been associated with increased risk of mortality and morbidity, many centers established protocols for glycemic control [6]. However, normoglycemia is not easy to establish, and barriers to widespread adoption of tight glucose control were many including an increased risk of severe hypoglycemia, a difficulty in achieving normoglycemia, as well as an increase in the resources and the workload for medical staff [6, 7]. Because of these issues and uncertainty about the balance of risks and benefits, tight glucose control is used infrequently by clinicians [8, 9].

Development of a closed loop glucose control system that automatically infuses insulin based on an automated algorithm that integrates a continuous glucose signal, could help overcome these obstacles and permit strict glycemic control without increasing the workload for the medical staff. This is the first study to examine the effectiveness of a closed loop glucose control system on intra- and post- operative glucose levels in patients undergoing open heart surgeries. Our hypothesis is that the automated protocol will allow a higher percentage of diabetic patients undergoing open heart surgery to remain in the glycemic corridor of 7.8–10 mmol.l^−1^ as compared to the conventional technique while avoiding hypoglycemic and hyperglycemic episodes.

## Patients and methods

This prospective randomized clinical trial was conducted at the American University of Beirut Medical Center and adheres to the applicable CONSORT guidelines for conducting and reporting clinical trials. This study was approved by the AUBMC Institutional Review Board (IRB ID #ANES.RK.03) and written informed consent was obtained from all subjects participating in the study. The study was registered prior to patient enrollment with clinicaltrials.gov (NCT #NCT03314272, Principal investigator Roland Kaddoum, date of registration: 19/10/2017). Diabetic patients undergoing cardiopulmonary bypass (CPB) surgery were approached and invited to participate in the study. Inclusion criteria included all diabetic patients (type I or II), aged between 40 and 75 years, ASA III and IV, admitted for CPB surgeries under general anesthesia. Patient’s refusal to consent, critically ill patients (ASA V or VI), pregnant patients, and emergency or lifesaving cases were excluded.

Upon admission, patients underwent standard preoperative anesthesia and surgery evaluation. After selecting and properly approaching the patients for our study, written informed consent was obtained in a private setting following our IRB guidelines. Demographic and physiological data as well as the presence of any diabetic complications such as nephropathy, retinopathy, and neuropathy were collected thereafter along with a set of baseline laboratory tests that were done routinely for each patient. In the induction room, an intravenous cannula was inserted by an experienced anesthesiologist or resident. A three-way stopcock was connected directly to the cannula. Standard anesthesia monitors (ECG, non-invasive blood pressure cuff, and pulse oximetry) were connected to the patient. A radial arterial line was inserted by the anesthesia team under sterile conditions using a 20-gauge Angio catheter. General anesthesia was then started. Afterwards, a baseline glucose level was taken from an arterial blood gas sample. At the induction phase, patients received propofol (1 mg.kg^−1^) and midazolam (0.05 mg.kg^−1^) or etomidate (0.3 mg.kg^−1^), in addition to intravenous lidocaine (1.5 mg.kg^−1^), rocuronium (1 mg.kg^−1^), and fentanyl (5 μg.kg^−1^) followed by rocuronium infusion and incremental doses of fentanyl throughout the surgery. Sevoflurane was used throughout the surgery for maintenance.

### Interventions

Patients were assigned to one of two groups; allocation was done according to a computer-generated table of random numbers created by the research coordinator. Proper concealment process was maintained using a sequentially numbered, opaque, sealed envelopes. The patient and the data collector were not aware of the allocation treatment.

In the control group, patients’ glucose levels were managed as instructed by the anesthesiologist in charge, according to the current practice using a sliding insulin scale protocol adopted at AUBMC for diabetic patients. It consists of giving intravenous Actrapid Insulin every 30 min based on blood glucose readings as follows: < 8.3 mmol.l^−1^: 0 units, 8.3–11.1 mmol.l^−1^: 2 units, 11.2–13.9 mmol.l^−1^: 4 units, 13.9–16.7 mmol.l^−1^: 6 units, 16.7–19.4 mmol.l^−1^: 8 units, > 19.5 mmol.l^−1^: 10 units.

In the experimental group, patients were treated using the Space Glucose Control System. The Glucose Control System (B. Braun Space® GlucoseControl—SGC System manufactured by B Braun Melsungen AG, Postfach 1120, D-34209 Melsung, and supported by Drogurie de L’union S.A.L.) is an automated system for glycemic control, that uses an already set algorithm (the enhanced model predictive control eMPC) to achieve a tight glucose window. It is a Class IIb medical device. The manufacturer, B. Braun, received the first CE marking in June 2004. The most recent renewal was in May 2013 [https://www.nice.org.uk/advice/mib17/chapter/technology-overview]. At the bottom of this system, a central user interface is connected with a touch screen interface in order to enter the data. Glucose readings as measured must be entered manually by the anesthesiologist via the touch screen display (the user interface). Based on this input, the system gives advice on the insulin infusion rate. The suggested insulin infusion rate is displayed on the screen but has to be manually confirmed before being automatically infused by the system.

In both groups, glucose measurements were obtained through an arterial blood gas sample taken every half an hour; insulin treatment was administered according to the allocation of the patient. The anesthesiologist was just asked to take an arterial blood gas sample from the patient every 30 min without knowing the purpose of the study so the anesthesiologist will be blinded to the intervention that was performed by the study research member. The range of glucose was recorded throughout the intraoperative period. The number of hypoglycemic, defined as blood glucose level less than 3.9 mmol.l^−1^ [10], and hyperglycemic, defined as blood glucose level greater than 11.1 mmol.l^−1^ [11], events were also recorded, as well as the first glucose reading in the post-cardiac surgery unit (CSU).

Hypoglycemic episodes, once detected, were treated promptly to provide a rise in blood glucose to a safe level to eliminate any potential harm. The intervention would include IV infusion of 100 to 200 ml of D5W over 1 to 3 min and repeating a blood glucose level after 15 min to reach a target blood sugar of at least 5.6 mmol.l^−1^. The same intervention would be repeated in case this level wasn’t reached. The patient would be withdrawn from the study after these two failed corrections and then would be treated more aggressively with an IV infusion of D10W.

As a primary outcome, we evaluated the percentage of patients who had a target glucose level between 7.8 and 10 mmol.l^−1^, and in addition, the percentage of patients who had at least one hypo- or hyper-glycemic events. Secondary outcomes included the analysis of the glucose levels trend intraoperatively (with a focus on the interaction between the group allocation and time), the percentage of patients who fell below 7.8 mmol.l^−1^, the percentage of patients who fell below 10 mmol.l^−1^, the percentage of patients whose glucose levels maintained within this safe range, and the percentage of patients who had any hypo- or hyper- glycemic event in the CSU, as well as the number of interventions by the anesthesiologist.

### Sample size calculation and statistical analysis

After conducting an internal pilot study on adult diabetic patients undergoing cardiac surgery with CPB, glucose levels of 20 patients were retrospectively studied. We found that 10 out of 20 patients fell out of the target glucose range (7.8–10 mmol.l^−1^), which corresponds to 50% of patients undergoing CPB surgery. The aim was to compare the fully automated algorithm to the routinely used scale. With a difference of 40%, Type I error of 5% and a Type II error of 20%, a power analysis revealed that at least 22 patients were needed in each group. To account for dropouts, 30 patients were recruited in each group.

Patients’ demographics were collected in both groups and compared using Chi square for categorical data, and student’s t-test for continuous data (Mann Whitney U test was used for variables with non-parametric data distributions).

The percentages of adequate glycemic control (≤ 7.8 mmol.l^−1^, ≤ 10 mmol.l^−1^, 7.8–10 mmol.l^−1^), hyperglycemia (> 11.1 mmol.l^−1^), and hypoglycemia (< 3.9 mmol.l^−1^) were presented as numbers (percentages) and Odds ratio (95% CI) and compared between patients of the two study groups using the Chi-Square test. Mixed model analysis was used to study the correlation between the study randomization arm and the time element on glucose levels. Data were analyzed using the Statistical Package for Social Sciences (SPSS Inc., Chicago, IL, USA) software (version 27). The clinical significance threshold was set at 0.017 for the primary outcomes (to account for the multiple primary outcomes using Bonferroni correction) and 0.05 for the secondary outcomes.

## Results

**Fig. 1:**
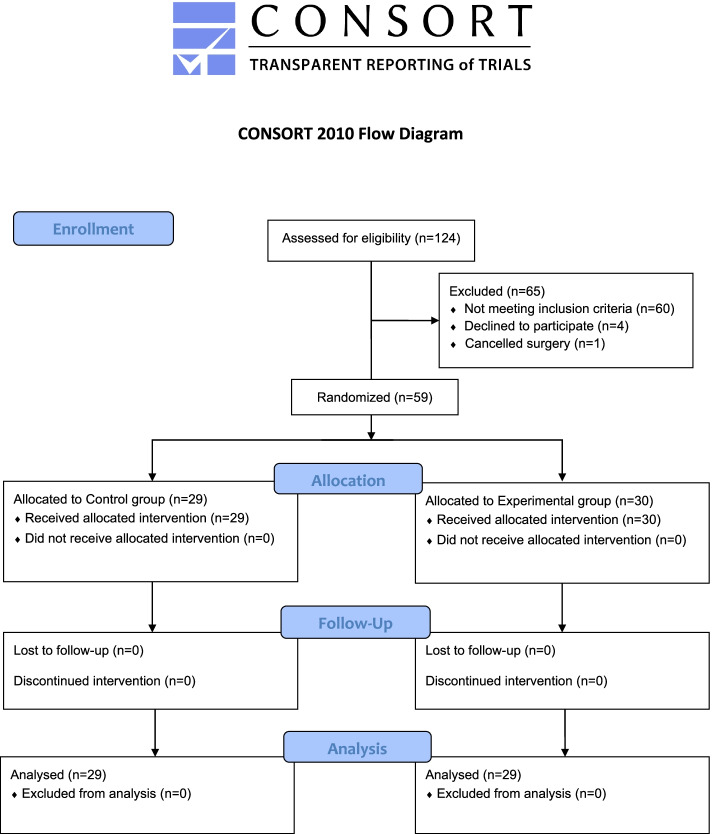
Consort flow diagram of patient recruitment

One hundred twenty-four patients were screened, out of which fifty-nine patients with diabetes mellitus type II were consented and randomized between May 2018 and February 2021. The reasons for exclusion are presented in the CONSORT flow diagram (Fig. 1). Baseline characteristics of the sample studied are depicted in Table 1. All patients were euvolemic and medically optimized, as they all undergone elective surgeries.

**Table 1:** Baseline characteristics of patients allocated to the control group (glucose levels managed using the routinely used sliding scale) versus patients allocated to the experimental group (glucose levels managed using the automated algorithm)

	Control (*n* = 29)	Experimental (*n* = 30)
Age	61.0 (9.3)	64.3 (7.7)
BMI	28.9 (5.2)	30.6 (5.1)
Gender		
Females	10 (34.5)	4 (13.3)
Males	19 (65.5)	26 (86.7)
ASA		
3	19 (65.5)	26 (86.7)
4	10 (34.5)	4 (13.3)
Diabetes complications		
Nephropathy	3 (10.3)	5 (16.7)
Nephropathy + Neuropathy	1 (3.4)	1 (3.3)
Nephropathy + Neuropathy + Retinopathy	1 (3.4)	0 (0)
None	24 (82.8)	24 (80)
Baseline glucose level	166 (55.2)	142.7 (35.8)
Surgery type		
CABG	17 (58.6)	20 (66.7)
CABG and valve repair	5 (17.2)	7 (23.3)
Valve repair	4 (13.8)	2 (6.7)
Aortic root replacement	2 (6.9)	1 (3.3)
Atrial septal defect repair	1 (3.4)	0 (0)
Cardioplegia type		
Saint-Thomas Cardioplegia Solution	15 (51.7)	21 (70)
Custodial Cardiplegia Solution	8 (27.6)	6 (20)
Delnido Cardioplegia Solution	5 (17.2)	3 (10)
None	1 (3.4)	0 (0)
Duration of aortic cross clamping	96.6 ± 48.2	97.2 ± 37.8
Cardiopulmonary bypass time	135.1 ± 60.3	128.8 ± 43.7

*CABG* Coronary Artery Bypass Graft

Patients’ glucose levels maintained between 7.8 and 10 mmol.l^−1^ throughout the surgery period and the percentage of time spent within this safety range were not significantly different between the two groups (Table 2). However, the proportion of patients who had their glucose levels below or equal to 7.8 mmol.l^−1^ for more than 95% of their surgical time were significantly higher in the experimental group compared to the control group (33.3% versus 13.8%, odds ratio 4.3 95% CI [1.1–17.8], *P* = 0.03). As well as the proportion of patients who had their glucose levels below or equal to 10 mmol.l^−1^ for more than 95% of their surgical time were significantly higher in the experimental group compared to the control group (83.3% versus 41.4%, odds ratio 7.1 [2.1–23.8], *P* = 0.001).

**Table 2:** Glucose levels maintained within different ranges over the surgery period and percentage of time spent with the glucose levels maintained between 7.8–10 mmol.l^−1^ and 6.7–10 mmol.l.^−1^

	Control (*n* = 29)	Experimental (*n* = 30)	Odds ratio 95% CI	*P* value
Glucose levels maintained ≤ 7.8 mmol.l^−1^ (more than 50% of the surgery time)			4.4 (1.4–13.8)	0.01
Yes	6 (20.7)	16 (53.3)		
No	23 (79.3)	14 (46.7)		
Glucose levels maintained ≤ 7.8 mmol.l^−1^ (more than 95% of the surgery time)			4.3 (1.1–17.8)	0.03
Yes	3 (13.8)	10 (33.3)		
No	26 (86.2)	20 (66.7)		
Glucose levels maintained ≤ 10 mmol.l^−1^ (more than 50% of the surgery time)			8.56 (1.7–43.2)	0.004
Yes	18 (62.1)	28 (93.3)		
No	11 (37.9)	2 (6.7)		
Glucose levels maintained ≤ 10 mmol.l^−1^ (more than 95% of the surgery time)			7.1 (2.1–23.8)	0.001
Yes	12 (41.4)	25 (83.3)		
No	17 (58.6)	5 (16.7)		
Glucose levels maintained between 7.8–10 mmol.l^−1^ (more than 50% of the surgery time)			1.9 (0.7–5.3)	0.29
Yes	11 (37.9)	16 (53.3)		
No	18 (62.1)	14 (46.7)		
Glucose levels maintained between 7.8–10 mmol.l^−1^ (more than 95% of the surgery time)			2.3 (0.6–5.6)	0.21
Yes	4 (13.8)	8 (26.7)		
No	25 (86.2)	22 (73.3)		
Glucose levels maintained between 6.7–10 mmol.l^−1^ (more than 50% of the surgery time)			2.9 (0.9–8.7)	0.06
Yes	14 (48.3)	22 (73.3)		
No	15 (51.7)	8 (26.7)		
Glucose levels maintained between 6.7–10 mmol.l^−1^ (more than 95% of the surgery time)			4.2 (1.3–14.0)	0.016
Yes	5 (17.2)	14 (46.7)		
No	24 (82.8)	16 (53.3)		
Percentage of time spent with glucose levels maintained between 7.8–10 mmol.l^−1^	35.1 (34.1)	37.1 (37.0)	-0.06^a^ (-0.6–0.4)	0.8
Percentage of time spent with glucose levels maintained between 6.7–10 mmol.l^−1^	44.3 (36.3)	60.6 (40.1)	-0.42 (-0.9–0.1)	0.11

*CI* Confidence interval of point estimate

^a^Hedges’ g is interpreted for continuous outcome

Patients who had at least one intraoperative hyperglycemic event were significantly higher in the control group compared to the experimental group, 17 (58.6%) vs. 5 (16.7%), odds ratio 7.1, 95% CI [2.1–23.8], *P* value < 0.001, respectively. In addition, the number of hyperglycemic events were significantly higher in the control group compared to the experimental group, 1 [0, 4] vs 0 [0, 0], *p* < 0.001, respectively.

We did not observe any remarkable intraoperative hypoglycemic event in both groups, but for one patient in the control group who had two hypoglycemic events, 2.7 mmol.l^−1^ and 3.5 mmol.l^−1^ at time stamps 120 min and 180 min respectively. The number of patients who developed at least one reading of glucose greater than or equal to 10 mmol.l^−1^ over their surgery period was significantly higher in the control group compared to the experimental group, *n* = 22 (75.9%) vs. *n* = 9 (30%), *P* = 0.001, respectively.

**Fig. 2:**
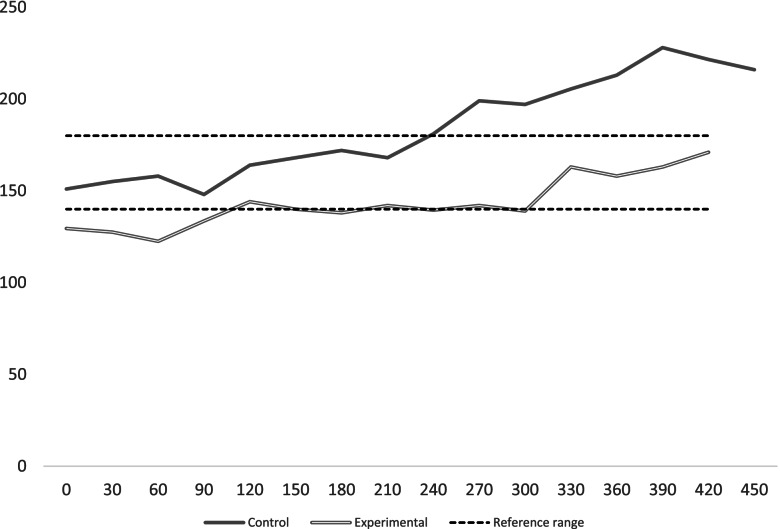
Average glucose concentration in the two groups over time

Mixed model analysis also showed that the fluctuations in average glucose levels over the surgical time were significantly different between the two groups. Figure 2 represents the average glucose levels at each time stamp over the surgery period for the two groups. The experimental group had lower glucose levels without falling below 6.7 mmol.l^−1^. To assess whether the differences between the intervention and control groups were different over time we tested an interaction term between the intervention and linear time, in addition, based on Fig. 2 where the average glucose level in the control group exceeded 10 mmol.l^−1^ at the 210 min time-point, and in order to facilitate interpretations, we also created a binary time variable, – split into two surgical duration categories, before and after 210 min time stamp – and tested its interaction with group allocation. Results showed that those in the treatment group had lower glucose levels over surgical time. The mixed model analysis with the binary time variables (Table 3) showed that glucose levels were significantly higher post the 210 min time stamp and that the automated protocol additionally decreased this elevation in glucose levels by 1.1 mmol.l^−1^ (interaction regression coefficient was –19, 95% CI [-29; -8], *P* value < 0.001).

**Table 3:** Mixed Model Analysis for the effect of the interaction between Allocation and Time on Glucose Levels

	Estimate	*P* value	95% Confidence Interval
First Model			
Allocation (Control/Experimental)	-21.2	0.037	-41.2 – -1.3
Time (minutes)	0.2	< 0.001	0.1 – 0.3
Interaction between Allocation and Time	-0.06	0.004	-0.1 – -0.02
Second Model (with a cutoff at 210 min)			
Allocation (Control/Experimental)	-26.2	0.009	-45.5 – -6.9
Time (Shorter/longer than 210 min)	50.1	< 0.001	33.8 – 66.4
Interaction between Allocation and Time	-18.9	< 0.001	-29.5 – -8.4

In the experimental group the intervention was limited to the preparation of the pump before the procedure start that is one intervention by the anesthesiologist compared to a median of 5 interventions needed for preparation and administration of insulin in the Control group (*P* < 0.001).

Upon arrival to the CSU, postoperative hyperglycemia was significantly higher in the control group compared to the experimental group (13 (46.4%) vs. 4 (13.3%), *P* = 0.009); the number of patients with postoperative glucose levels maintained between 7.8 and 10 mmol.l^−1^ was significantly higher in the experimental group compared to the control group, 15 (50%) vs. 5 (17.9%) with *P* = 0.013, respectively; postoperative hypoglycemic events were not observed in any of the patients.

One patient in the control group passed away during the surgery (the postoperative glucose level of this patient was left missing since it is the only missing value for this variable), and another patient in the control group died three days after the surgery. These events were not related neither directly nor indirectly to any of the study interventions.

### Exploratory analysis

The original assumption of our primary outcome safety zone (7.8–10 mmol.l^−1^) was based on a conservative pilot study. However, since we did not encounter any hypoglycemic event in the experimental group and only two hypoglycemic events in the control group, a wider safety zone was sought taking into consideration the minimum average glucose level at each time point (Fig. 2). Therefore, we also analyzed the number of patients whose glucose levels were maintained between 6.7–10 mmol.l^−1^.

This additional analysis showed that patients’ glucose levels maintained intraoperatively between 6.7 mmol.l^−1^ and 10 mmol.l-1 were significantly higher in the experimental group compared to the control group, 14 (26.7%) vs 5 (17.2%) with a *P* = 0.025, respectively.

## Discussion

The literature is dense with studies reporting results of tight glucose control in the ICU and most of them were able to confirm the hypothesis that a tight glucose control decreases mortality and morbidity in critically ill patients [1, 6, 7, 12–15]. However, to our knowledge, very few studied tight glucose control in surgical patients [2, 16] with our study being the first to verify this hypothesis in open heart surgical patients using an automated glucose control system.

Our study showed that the use of the automated Glucose Control System from B.Braun maintained patients within a safe glucose window (7.8 – 10 mmol.l^−1^) with a very low incidence of hyperglycemic events and no hypoglycemic events in surgical patients undergoing open-heart procedures as compared to manual control that showed a similar number of patients within a safe glucose window (7.8 – 10 mmol.l^−1^), but with remarkably higher hyperglycemic events. Maintaining glucose levels within a target range of 7.8 – 10 mmol.l^−1^ using the Glucose Control System successfully met the recommendations by the Society for Ambulatory Anesthesia (SAMBA) and the Society of Thoracic surgery: Intraoperative blood glucose levels < 10 mmol.l^−1^, while avoiding hypoglycemia [17, 18]. The non-significant difference in the proportion of patients who had their glucose levels maintained between 7.8 and 10 mmol.l^−1^ between the two groups intraoperatively is related to significantly lower glucose levels – below 7.8 mmol.l^−1^ – in the experimental group and higher glucose levels – above 10 mmol.l^−1^ – in the control group. Therefore, the automated system performed better in avoiding hyperglycemic episodes without significant hypoglycemia as evidenced by a low proportion of patients with hyperglycemic episodes in the experimental group. In addition, we did not encounter any hypoglycemic event in the experimental group with only one patient with two events in the control group that was rapidly corrected.

In the post-surgical setup, a recent systematic review was conducted on patients who underwent cardiac surgery and received insulin therapy over 5 days post-surgery for glycemic control using continuous insulin infusion algorithm. Similar to our study, it showed that using an automated system achieved more regulated levels of blood sugar compared to bolus administration [2].

Surgery and anesthesia cause stress response and produce significant neurophysiological changes, which can lead to significant hyperglycemia. This latter can impair leukocyte function causing immunosuppression and subsequent infection as well as overall mortality [19]. On the other hand, intraoperative hypoglycemia (blood glucose level below 3.9 mmol.l^−1^) predisposes the patient to brain injury, which sometimes might be fatal [20], mandating a prompt management of hypoglycemic events.

Noteworthy to mention, the automated Space Glucose Control System reduces the time needed for intervening by the anesthesiologist by minimizing the time required for preparation and administration of insulin. In our study, this was reflected by the significantly lower number of interventions in the experimental group compared to the control group. Indeed, the number of interventions in the experimental group is notably limited to the setup and preparation of the pump before the surgery starts.

There is no consensus on a precise intraoperative blood glucose level, except for some recommendations concerning glycemic control from diverse societies. Since there is no high-level evidence research that succeeded in implementing a well-defined range for intraoperative glucose levels maintenance [18], we relied on general principles suggested by the societies. Our study confirmed the safety of these principles.

In our study, the pump settings were calibrated to 7.8–10 mmol.l^−1^ to prevent any hypo- or hyperglycemic event. The results showed that this target successfully kept the patients within a safe range with the average glucose levels of a minimum of 7.4 (2) mmol.l^−1^ (at time stamp 60 min) and a maximum of 8.9 (0.5) mmol.l^−1^ (at time stamp 390 min). Further analysis showed that these settings were keeping the patients between a lower safety level of 6.7 and a maximum of 10 mmol.l^−1^, suggesting the possibility of widening the safety range from 7.8–10 mmol.dl^−1^ to 6.7–10 mmol.dl^−1^. Thereby, we showed that an automated system set at a range of 7.8–10 mmol.l-1 actually keeps glucose levels of most patients levels between 6.7–10 mmol.l^−1^.

Our results showed that the longer the surgery time was (> 210 min), the more the patients were prone to hyperglycemic events (> 11.1 mmol.l^−1^) exceeding the upper limit of 10 mmol.l^−1^. These results were confirmed using the mixed model analysis that clearly showed increased benefits of the automated system in surgeries lasting more than 210 min.

Our study has its limitations. Blinding of the glucose control system operator was not possible due to the design of our study. Shorter time intervals were not possible because the pump’s settings were already automated. The pump was automated according to a specific algorithm that cannot be changed according to our findings after initiating the study. In addition, some of the interesting results in our study resulted from secondary outcomes and post hoc exploratory analysis. Further research using closed loop could benefit from our results to conduct future studies.

## Conclusions

This study showed that using an automated glucose control pump in diabetic patients undergoing open heart surgeries maintained most of the patients within a predefined glucose range with a very low incidence of hyperglycemic events and no incidence of hypoglycemic events, particularly in longer surgeries. In addition, it is less laborious in terms of interventions by the anesthesiologist. Future studies are needed to explore the safety of other automated ranges than the one we studied.

## Data Availability

The datasets used and/or analysed during the current study are not publicly available because the publicity policy is not yet generated by our institutional review board but are available from the corresponding author on reasonable request.

## Abbreviation

CSU: Post-cardiac surgery unit
